# A rare case of scedosporium apiospermum osteomyelitis in an immunocompetent patient

**DOI:** 10.1016/j.idcr.2024.e01929

**Published:** 2024-01-30

**Authors:** Aayushi J. Rajani, Darshankumar Raval, Rohit Chitale, Ravindra Durvasula, Justin Oring, Ross Powers

**Affiliations:** Mayo Clinic Florida, Infectious Disease Department, United States

**Keywords:** Scedosporium Apiospermum, Dual antifungal therapy, Voriconazole, Caspofungin

## Abstract

Scedosporium, a widespread filamentous fungus found in diverse environments, has experienced a rise in cases due to escalating malignancies and chronic immunosuppression. Clinical manifestations span mycetoma, airway involvement, and various infections, with osteomyelitis being a notable complication. We present a case of a 77-year-old female initially displaying cutaneous Scedosporium signs, which progressed to osteomyelitis. The patient, with a history of trauma, chronic low dose steroid use, and underlying conditions, presented with a foot injury caused by her dog. Despite initial management, worsening symptoms led to the identification of Scedosporium. A comprehensive approach involving debridement, antimicrobial therapy, and reduction of immunosuppression resulted in clinical improvement. The rarity of zoonotic transmission, diagnostic challenges, and antifungal efficacy are also discussed. The patient's positive trajectory emphasizes early diagnosis, targeted treatment, and vigilance in managing immunosuppression. An adaptable treatment protocol is proposed based on risk factors. Considering the rising opportunistic fungal infections and delayed culture results, initiating empirical antifungals based on clinical judgment and regional prevalence is vital for favorable outcomes.

## Introduction

Scedosporium is a filamentous fungus which is found worldwide in soil, sewage, and polluted waters [1]. A recent rise in cases of Scedosporium has been seen due to an increase in the number of malignancies as well as the number of people living with chronic immunosuppression. Its clinical manifestations can be categorized into four distinct conditions: mycetoma, saprobic involvement of the airways, localized sinopulmonary or extrapulmonary infections, and disseminated infections [2]. Even though it is mostly an opportunistic infection, Scedosporium osteomyelitis as a complication following a deep puncture injury is seen in both immunocompromised and immunocompetent hosts [3]. Voriconazole is considered the first-line treatment with the most potent in vitro activity noted in various studies. Caspofungin was seen to be the most active echinocandin in the same studies [2]. We present a case of extrapulmonary localized infection in a 77-year-old female who originally presented with possible cutaneous manifestations of *Scedosporium apiospermum* that progressed to involve the bone, leading to osteomyelitis.

## Case presentation

A 77-year-old female presented to the clinic with the chief complaint of a recent injury to the foot. Her dog stepped on it, leading to bruising and pain on her left dorsum. Her past medical history is significant for polymyalgia rheumatica, for which she is on chronic low-dose prednisone therapy, a mechanical mitral valve due to which she is on chronic anticoagulation, CKD, and a well-differentiated SCC on her chest. In addition to that, her dog had also stepped on her right foot five months ago, leading to the fracture of her second metatarsal base, which is well healed at present.

On general examination, the patient was vitally stable. On musculoskeletal examination, her left foot was tender with bruising; she was still weight bearing without difficulty. All routine investigations were normal. To rule out fractures an X-ray was taken, and she was found to have a nondisplaced second metatarsal fracture, which was managed conservatively.

On her follow-up visit a week later, the puncture wound was now draining exudate. There was associated excoriation at the site along with pain and redness all over the dorsum. Wound cultures with a swab were sent due to the suspicion of cellulitis, and doxycycline was started empirically, as mentioned in the timeline shown in Fig. 1.

The cultures grew *Enterococcus faecalis*, and doxycycline was continued as she was responding well to it with almost complete resolution of the swelling and marked improvement in pain. The puncture site also started to scab over.

However, on her next follow-up visit, 2 weeks later, she complained that since the completion of doxycycline, she had been noticing increased erythema and mild edema along with a couple of lesions that were draining yellow purulent fluid with mild pain but no fever. Repeat cultures were sent, and she was empirically started on a course of clindamycin. The fungal cultures showed growth of *Candida parapsilosis* and Scedosporium species complex. The possibility of it being a contaminant was high as it was a superficial swab culture taken from the wound. Hence it was disregarded, and clindamycin was continued.

There was no improvement noted on clindamycin, so Levofloxacin was added to try to provide better coverage for gram-negative aerobes and streptococcus.

**Image 1:**
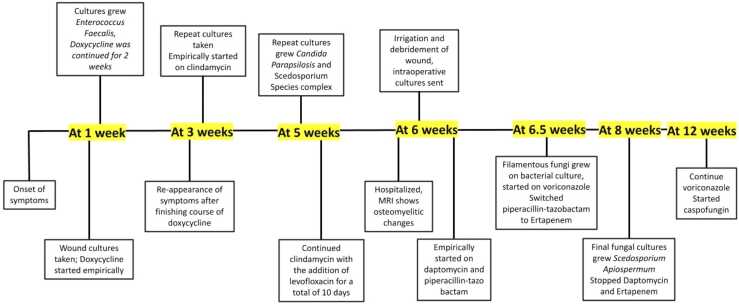
Timeline highlighting all the significant events that occurred.

**Image 2:**
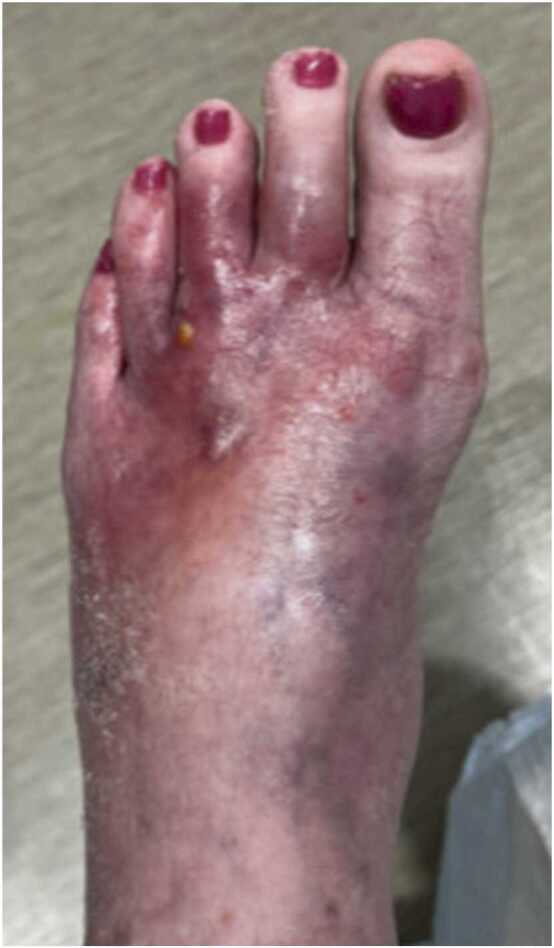
Several small pustules containing purulent material as well as non-blanchable punctate areas of erythema with tenderness to palpation.

On her follow-up visit a week later, she was hospitalized as her left foot fracture was still not healing even 1.5 months after the injury. She was also evaluated for osteomyelitis. Foot seen in Image 2.

The MRI showed “septic arthritis with evidence of early osteomyelitis along the lateral aspect of the second metatarsal head with cellulitis and phlegmon of the dorsum of the foot.” Incomplete or delayed healing of the fracture was also seen. As she was hemodynamically stable, it decided to withhold starting any new antibiotics until bone biopsies were obtained so their diagnostic yield could be increased.

A thorough irrigation and debridement was conducted, intraoperative cultures were sent, and she was empirically started on IV daptomycin and piperacillin-tazobactam postoperatively. The prednisone dose was also gradually tapered down to aid in clearing the infection. The cultures grew filamentous fungi on the bacterial culture, and we started a course of voriconazole.

The final culture results showed growth of only *Scedosporium apiospermum*. The specimen was sent to University of Texas at San Antonio for antimicrobial susceptibility testing. The results show minimum inhibitory concentrations (MICs) for the following: amphotericin 8 mcg/ml, posaconazole 2 mcg/ml, voriconazole 2 mcg/ml, isavuconazole > 16, and caspofungin 0.125 mcg/ml.

On her follow-up visits in the subsequent weeks, she was seen to improve with signs of early re-epithelialization, but she still had some amount of persistent swelling with bruising and associated discoloration of the toes and the foot. Given only mild improvement and high MIC to voriconazole, it was decided to add caspofungin IV. We made sure to keep a close watch on her during follow-up to prevent complications such as a recurrence leading to amputation.

## Discussion

We hypothesize that the patient’s dog stepped on her foot leading to the traumatic inoculation of *Scedosporium apiospermum* from soil [4]. Combined with the immunosuppression as well as the malignancy, it led to infection of the wound with subsequent osteomyelitis [3].

When Scedosporium Apiospermum affects the eyes, trauma is the most important factor to consider. History of corticosteroid use in the past is also known to be a risk factor [4]. Similarly, our patient has a history of trauma as well as chronic low dose steroid use.

Lamaris et al. noted that the incidence of Scedosporium was 0.82 cases per 10,000 inpatient days in 1933–1988 and 1.33 cases per 100,000 patient-inpatient days in 1999–2005 [5]. Even though it is very rare, an increased incidence of opportunistic infections caused by Scedosporium is being seen in patients on chronic steroid therapy, immunosuppressants, antineoplastics, broad-spectrum antibiotics, and people with malignancies [6], [7]. Similarly, our patient was on chronic low dose prednisone therapy since 5 + years and had a well differentiated SCC and hence there was increased host susceptibility to acquire this infection.

Scedosporium apiospermum is known to cause human infection either after traumatic subcutaneous implantation of its conidia or after inhalation in cases with near-drowning. Disseminated infections can also occur in immunocompetent patients after near-drowning events. It is a differential to keep in mind if pneumonia or brain abscess develops in a patient with history of near-drowning [8]. Skin infections are frequently linked to traumatic inoculations caused by contamination of the affected areas with soil or plant debris, as in our patient [9].

There is only one other case reported which has zoonotic transmission involving trauma, a man developed similar looking cutaneous manifestations post a dog bite for which he didn’t seek early medical attention. He also had pustules which were draining purulent material, like our patient, which then scabbed over as time progressed [10]. Other than that no other cases of zoonotic transmission have been reported as far we can see.

The overall mortality rate seen for Scedosporium is around 50 %, and it increases to about 90 % in disseminated diseases [7], [11]. The patient did well on the prescribed course and was kept under close follow-up to prevent any complications. Voriconazole is the antifungal with the greatest efficacy against *Scedosporium apiospermum* [11]. It is often used as a monotherapy to treat patients. When it was compared to Amphotericin B, Voriconazole was seen to be associated with a better trend for survival [12], [13]. Poor activity of Amphotericin against Scedosporium is well documented and using it in a combination therapy with Voriconazole offers no advantage [5]. Interestingly, studies have shown that Amphotericin doesn’t have any in vitro activity against Scedosporium apiospermum as it has intrinsic resistance to it [14]. In other studies involving various echinocandins it was seen that Caspofungin was the most active against *Scedosporium apiospermum* out of all the echinocandins tested [2]. Dual therapy was preferred as the patient was immunosuppressed and there is a high risk of mortality associated with these cases. We also didn’t see adequate response to using Voriconazole as a monotherapy. There have been other patients also who needed combined therapy to help improve outcomes due to their preexisting comorbidities [15].

There are still several challenges associated with diagnosing it and treating it. It requires labor-intensive histopathology as well advanced non-culture based systems at the diagnosing center. Due to treatment resistance, new antifungal drugs are being developed to treat Scedosporium species. Since Scedosporium infection has high risk of becoming severe, early diagnosis, antifungal therapy, surgical excision of necessary and reversal of immunosuppression when possible is recommended [16].

The patient has been seen to improve on follow up over the past 5 months. She is still being followed up.

**Image 3:**
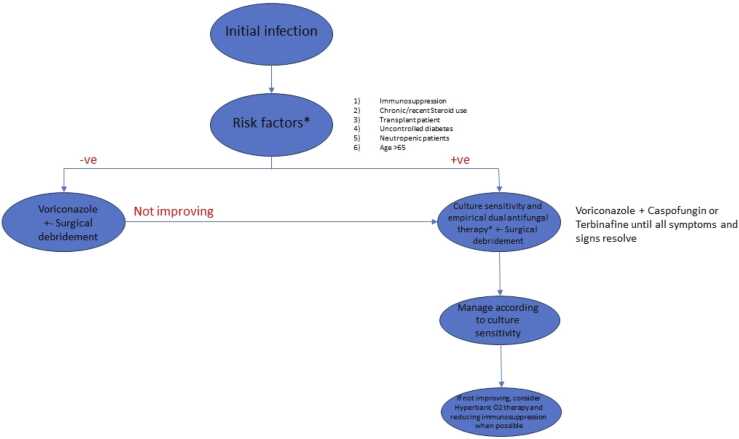
suggested treatment protocol.

As mentioned in the flow chart, selection of the regimen depends on the risk factors like old age, immunosuppression, prolonged neutropenia, transplant patients etc. If risk factors are present then we should treat the patient with dual antifungals regimen including Voriconazole and Caspofungin or Terbinafine. If there are no risk factors, we can choose monotherapy with Voriconazole. Surgical debridement should be done whenever necessary [12].

Because of the rise in the incidence of opportunistic fungal infections in the recent past and the fact that fungal cultures take a long time to result, empirical antifungals should be started based on clinical suspicion and the regional prevalence to ensure good outcomes in patients.

## Ethical approval

Not required as patient identity isn’t revealed.

## Consent

Taken.

## Author contribution

Aayushi J Rajani: Study Design, Data Collection, Data Analysis, Writing, Editing, Darshankumar Raval: Data Analysis, Editing, Rohit Chitale: Data Analysis, Ravindra Durvasula: Data Analysis, Editing, Justin Oring: Data Analysis, Editing, Ross Powers: Data Analysis, Editing.

## Funding

No funding.

## Conflict of interest

Nothing to declare.
